# Efficacy and safety of guselkumab in biologic-naïve patients with active axial psoriatic arthritis: study protocol for STAR, a phase 4, randomized, double-blinded, placebo-controlled trial

**DOI:** 10.1186/s13063-022-06589-y

**Published:** 2022-09-05

**Authors:** Dafna D. Gladman, Philip J. Mease, Paul Bird, Enrique R. Soriano, Soumya D. Chakravarty, May Shawi, Stephen Xu, Sean T. Quinn, Cinty Gong, Evan Leibowitz, Denis Poddubnyy, Lai-Shan Tam, Philip S. Helliwell, Arthur Kavanaugh, Atul Deodhar, Mikkel Østergaard, Xenofon Baraliakos

**Affiliations:** 1 Centre for Prognosis Studies in The Rheumatic Diseases, Toronto Western Hospital, Toronto, ON Canada; 2 Swedish Medical Center/Providence St. Joseph Health and University of Washington, Rheumatology Research, Seattle, WA USA; 3 University of New South Wales, Randwick, NSW Australia; 4 Hospital Italiano de Buenos Aires, Buenos Aires, Argentina; 5 Janssen Scientific Affairs, LLC, Horsham, PA USA; 6 Drexel University College of Medicine, Philadelphia, PA USA; 7 Immunology Global Medical Affairs, Janssen Pharmaceutical Companies of Johnson & Johnson, Horsham, PA USA; 8 Janssen Research & Development, LLC, Spring House, PA USA; 9 Charité– Universitatsmedizin Berlin, Berlin, Germany; 10 The Chinese University of Hong Kong, Hong Kong, China; 11 University of Leeds, School of Medicine, Leeds, UK; 12 University of California at San Diego, La Jolla, CA USA; 13 Oregon Health & Science University, Portland, OR USA; 14 University of Copenhagen, Copenhagen, Denmark; 15 Rheumazentrum Ruhrgebiet, Ruhr-University Bochum, Herne, Germany

**Keywords:** Psoriatic arthritis, Axial psoriatic arthritis, Sacroiliac joint, Spine inflammation, MRI, Randomized controlled trial, Guselkumab, IL-23p19

## Abstract

**Background:**

Axial involvement constitutes a specific domain of psoriatic arthritis (PsA). Interleukin (IL)-23 inhibitors have demonstrated improvement in axial PsA (axPsA) symptoms, but have not shown efficacy in treating ankylosing spondylitis (AS), suggesting differences in axPsA processes and treatments. In a post hoc, pooled analysis of patients with investigator- and imaging-confirmed sacroiliitis in two phase 3, randomized, placebo-controlled studies (DISCOVER-1 and DISCOVER-2), patients treated with guselkumab, an IL-23p19 inhibitor, had greater axial symptom improvements compared with placebo. Confirmatory imaging at baseline was restricted to the sacroiliac (SI) joints, occurred prior to/at screening, and was locally read.

**Methods:**

The STAR study will prospectively assess efficacy outcomes in PsA patients with magnetic resonance imaging (MRI)-confirmed axial inflammation. Eligible, biologic-naïve patients with PsA (*N* =  405) for ≥ 6 months and active disease (≥ 3 swollen and ≥ 3 tender joints, C-reactive protein [CRP] ≥ 0.3 mg/dL) despite prior non-biologic disease-modifying antirheumatic drugs, apremilast, and/or nonsteroidal anti-inflammatory drugs will be randomized (1:1:1) to guselkumab every 4 weeks (Q4W); guselkumab at week (W) 0, W4, then every 8 weeks (Q8W); or placebo with crossover to guselkumab at W24, W28, then Q8W. Patients will have Bath Ankylosing Spondylitis Disease Activity Index (BASDAI) score ≥ 4, spinal pain component score (0–10 visual analog scale) ≥ 4, and screening MRI-confirmed axial involvement (positive spine and/or SI joints according to centrally read Spondyloarthritis Research Consortium of Canada [SPARCC] score ≥ 3 in ≥ 1 region). The primary endpoint is mean change from baseline in BASDAI at W24; multiplicity controlled secondary endpoints at W24 include AS Disease Activity Score employing CRP (ASDAS), Disease Activity Index for PsA (DAPSA), Health Assessment Questionnaire – Disability Index (HAQ-DI), Investigator’s Global Assessment of skin disease (IGA), and mean changes from baseline in MRI SI joint SPARCC scores. Centrally read MRIs of spine and SI joints (scored using SPARCC) will be obtained at W0, W24, and W52, with readers blinded to treatment group and timepoint. Treatment group comparisons will be performed using a Cochran-Mantel-Haenszel or chi-square test for binary endpoints and analysis of covariance, mixed model for repeated measures, or constrained longitudinal data analysis for continuous endpoints.

**Discussion:**

This study will evaluate the ability of guselkumab to reduce both axial symptoms and inflammation in patients with active PsA.

**Trial registration:**

This trial was registered at ClinicalTrials.gov, NCT04929210, on 18 June 2021.

Protocol version: Version 1.0 dated 14 April 2021.

**Supplementary Information:**

The online version contains supplementary material available at 10.1186/s13063-022-06589-y.

## Background

Substantial proportions of patients with psoriatic arthritis (PsA) develop inflammation of the sacroiliac (SI) joints and/or spine, both early (5–28%) and particularly later (25–70%) in the disease process; thus, axial PsA (axPsA) constitutes an important disease domain in PsA [1–9]. While axial involvement is considered part of the spectrum of axial spondyloarthritis (axSpA), where ankylosing spondylitis (AS) is the prototypical presentation, a large body of literature suggests that axial involvement in patients with PsA may be a distinct presentation from AS or axSpA, with differing clinical manifestations, genetic markers, and radiographic findings [8, 10]. About 20% of patients with PsA have the major histocompatibility class one surface antigen human leukocyte antigen (HLA)-B27, which has been associated with axial involvement and more severe disease [11]. Axial PsA has also been associated with HLA-B08, B38, and B39 [12]. Axial involvement has been associated with significantly worse disease across multiple clinical measures, including more severe skin manifestations, worse nail psoriasis, higher likelihood of enthesitis, higher tender joint counts, and lower likelihood of achieving minimal disease activity (MDA) [13].

To date, only one dedicated prospective randomized clinical trial has evaluated axPsA using imaging assessments in a subset of patients. The phase 3b study evaluated the efficacy and safety of secukinumab, an inhibitor of interleukin (IL)-17A, a downstream effector cytokine in the IL-23/Th17 pathway implicated in the pathogenesis of PsA [14]. A separate analysis (post-hoc) from two phase 3 multicenter, double-blind, placebo-controlled studies demonstrated that ustekinumab, an anti-IL-12/IL-23 monoclonal antibody, showed significant improvements in axial signs and symptoms of PsA [9]. However, these findings have not been replicated among patients with AS, which further suggests differential disease processes for axial involvement [15].

Guselkumab, an IL-23 inhibitor that specifically binds the p19 subunit, has been approved for the treatment of adults with active PsA worldwide, based on two phase 3 studies, DISCOVER-1 (NCT03162796) and DISCOVER-2 (NCT03158285), conducted in patients with active PsA [16, 17]. A post hoc analysis of a DISCOVER-1 and DISCOVER-2 study subset of PsA patients with investigator-confirmed sacroiliitis (radiograph or magnetic resonance imaging [MRI]) showed significant and robust improvement in axial symptoms of PsA with both guselkumab 100 mg every 4 weeks (Q4W) and every 8 weeks (Q8W) treatment, as assessed by mean changes from baseline in the Bath Ankylosing Spondylitis Disease Activity Index (BASDAI) and AS Disease Activity Score employing C-reactive protein (CRP) (ASDAS) and by achievement of ≥50% improvement in BASDAI (BASDAI50) and ASDAS responses [18]. In these studies, confirmatory imaging at baseline was restricted to the SI joints, occurred prior to or at screening as confirmed by the investigator, and was locally read.

MRI of the SI joints and spine are the only feasible instrument to objectively measure treatment efficacy on the target organ in axPsA patients. Thus, the STAR study will prospectively evaluate the efficacy and safety of both guselkumab Q4W and Q8W in biologic-naive PsA patients with axial involvement confirmed by centrally read imaging (Additional file 1). Improvements in clinical axial symptoms and objective reduction in axial inflammation of the spine and the SI joints using MRI will be assessed. Although previous studies have demonstrated the efficacy of guselkumab in adults with active PsA, a placebo control was selected for STAR to establish the effects of guselkumab in this subpopulation of PsA patients with axial disease. The placebo selected for this study is identical in appearance to guselkumab.

## Methods

**Fig. 1:**
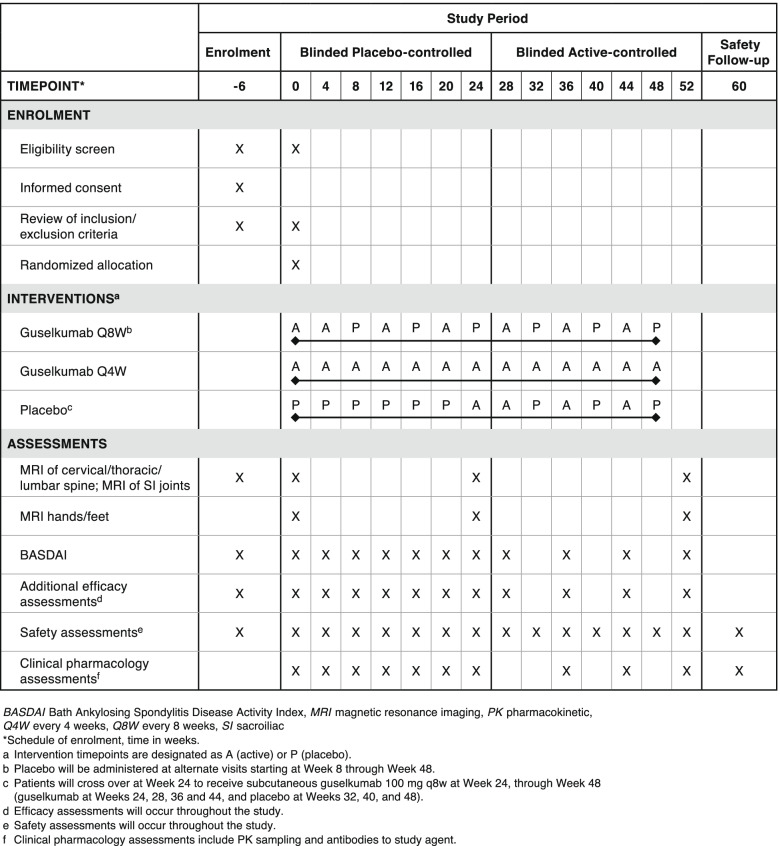
Standard protocol items: recommendation for interventional trials (SPIRIT) figure: trial visits and assessments.

This is a randomized, double-blind, placebo-controlled, parallel, multicenter, interventional study in biologic-naive patients with axPsA (Fig. 1; Standard protocol items: recommendation for interventional trials (SPIRIT) checklist is provided as Additional file 2 ). Patients will be recruited at private clinics and hospitals across global regions including Asia, Australia, Europe, North America, and South America. A listing of study sites can be found at https://clinicaltrials.gov/ct2/show/NCT04929210?term=CNTO1959PSA4002&draw=2&rank=1.

This study protocol follows the SPIRIT reporting guidelines [19].

### Objectives

An overview of objectives and endpoints is provided in Table 1. The primary study objective is to evaluate the efficacy of guselkumab treatment in patients with active axPsA in reducing axial symptoms assessed through the primary endpoint of change from baseline in BASDAI at week 24 (Table 1). The major secondary objectives are to evaluate the efficacy of guselkumab in treating axial symptoms utilizing additional outcome measures, including reduction in axial inflammation as assessed by MRI of the spine and/or SI joints, other signs and symptoms of PsA including skin psoriasis, and patient-reported outcomes. Among the major secondary endpoints are the changes from baseline in Spondyloarthritis Research Consortium of Canada (SPARCC) score for MRI SI joints and MRI spine at week 24 (for patients with positive MRI of the SI joints and spine, respectively, at baseline; Table 1). Efficacy will be evaluated through 1 year. Additional objectives include evaluating the safety of guselkumab in patients with axPsA through week 60, as well as the pharmacokinetics (PK) and immunogenicity of guselkumab in these patients. Patients will be monitored for adverse events (AEs) throughout the study.

**Table 1:** Study objectives and endpoints

Objectives	Endpoints
Primary	
• To evaluate the efficacy of guselkumab treatment in patients with active PsA axial disease by assessing reduction in axial symptoms	Mean change from baseline in BASDAI at week 24^a^
Major secondary	
• To evaluate the efficacy of guselkumab on additional measures of axial symptoms, reduction in axial inflammation, and other signs and symptoms of PsA, psoriasis, and patient well-being	Mean change from baseline at week 24 in: • ASDAS^a^ • DAPSA score^a^ • HAQ-DI score^a^ • SPARCC score for MRI SI joints (among patients with positive MRI of SI joints at baseline)^a^ • SPARCC score for MRI spine (among patients with positive spinal MRI at baseline) At week 24, proportion of patients achieving: • BASDAI50 response • ASDAS clinically important improvement (change ≥ 1.1) • ASDAS major improvement (change ≥ 2.0) • ASDAS inactive disease (score < 1.3) • ASAS40 response • IGA 0/1 response (among patients with ≥ 3% body surface area affected with psoriasis involvement at baseline)^a^
Other secondary	
• To evaluate the safety of guselkumab in patients with active PsA	For the duration of the study, through week 60: • Frequency and type of AEs, SAEs, AEs leading to discontinuation of study intervention, infections, and injection-site reactions • Frequency of laboratory abnormalities (chemistry, hematology) maximum toxicity (Common Terminology Criteria for Adverse Events [CTCAE 5.0]) grades
• To evaluate the PK and immunogenicity of guselkumab in patients with active PsA	Through week 60: • Mean/median serum guselkumab concentrations over time • Incidence of antibodies to guselkumab
Additional assessments	
Mean change from baseline through week 52 in: • CAN-DEN score for spine MRI (among patients with baseline CAN-DEN score ≥ 3) • OMERACT PsAMRIS score for MRI of the hands and feet (exploratory analysis in a subset of patients) by visit^b^	

*AE* adverse event, *AS* ankylosing spondylitis, *ASAS40* ≥40% improvement in Assessment of SpondyloArthritis international Society response criteria, *ASDAS* AS Disease Activity Score utilizing C-reactive protein, *BASDAI* Bath Ankylosing Spondylitis Disease Activity Index, *BASDAI50* ≥50% improvement in Bath Ankylosing Spondylitis Disease Activity Index score, *CAN-DEN* Canada-Denmark, *CTCAE 5.0* Common Terminology Criteria for Adverse Events, *DAPSA* Disease Activity Index for Psoriatic Arthritis, *HAQ-DI* Health Assessment Questionnaire – Disability Index, *IGA* Investigator’s Global Assessment, *MRI* magnetic resonance imaging, *OMERACT* Outcome Measures in Rheumatology, *PK* pharmacokinetics, *PsA* psoriatic arthritis, *PsAMRIS* Psoriatic Arthritis MRI Scoring System, *SAE* serious adverse event, *SI* sacroiliac, *SPARCC* Spondyloarthritis Research Consortium of Canada

^a^Multiplicity controlled endpoints that have been identified as important and assess different attributes of disease

^b^For this study, the investigator will select the most inflamed hand and the most inflamed foot; the selected hand and foot will be assessed by MRI at baseline, week 24, and week 52

The statistical analysis plan includes database locks at weeks 24 and 60. Primary and major secondary outcomes will be evaluated using data from the week 24 database lock. Post-week 24 data will subsequently be analyzed to examine the maintenance and trajectory of response through 1 year.

Serum samples will be collected at regular intervals for PK and immunogenicity assessments. Some HLA-B alleles have been shown to confer higher risk of developing axial involvement in patients with PsA; therefore, the HLA-B allele status will be determined for all patients. HLA-B27 status, associated with increased risk for developing axial PsA early in the disease course, will be used for stratification purposes in statistical analyses.

Biomarker assessments will be made to examine the biologic response to treatment and to identify biomarkers that are relevant to guselkumab treatment and/or PsA, where local regulations permit. Assessments (detailed below) will include the evaluation of relevant biomarkers in serum, plasma, and whole blood collected.

### Assessments

Disease activity will be assessed using the BASDAI. The BASDAI, scored from 0–10, is a patient-reported instrument evaluating the following six symptoms on a visual analog scale (VAS) (0–10 cm, 0 = none, 10 = very severe): fatigue, spinal pain, peripheral joint pain, pain at entheseal sites, severity of morning stiffness, and duration of morning stiffness [20, 21].

The ASDAS is a composite instrument, originally developed for patients with AS, that includes measures of back pain, duration of morning stiffness, patient global assessment, peripheral pain and swelling, and CRP [22]. The results of the post-baseline CRP measurements performed by the central laboratory will be blinded to the investigative sites. PsA disease activity will be assessed using the Disease Activity Index for Psoriatic Arthritis (DAPSA) [23], and the Investigator’s Global Assessment (IGA) [24] documents the investigator’s assessment of the patient’s psoriasis at a given timepoint. The functional status of the patient will be assessed by the Health Assessment Questionnaire-Disability Index (HAQ-DI) [25].

MRIs will be utilized to objectively assess reduction in axial inflammation. Centrally read MRIs of the spine and SI joints will be obtained at week 0, week 24, and week 52, with readers blinded to treatment group and timepoint. MRI scoring methods are summarized in Table 2. The SPARCC scoring system for MRI spine will be applied to the discovertebral unit, which is defined as the region between 2 imaginary lines drawn through the middle of adjacent vertebrae and including adjacent vertebral end plates with the intervening disc. All 23 discovertebral units are scored to yield the Spine Total score. Each MRI lesion is assessed on 3 consecutive sagittal slices, with additional points for “depth” and high “intensity” of the lesion [26, 27]. For the SI joint (among the patients with a positive MRI of the SI joint at baseline), the SPARCC method focuses on the cartilaginous portion of the SI joint, and documents presence (score of 1) versus absence (score of 0) of bone marrow edema in each SI joint quadrant (defined according to a vertical axis through the joint cavity and a horizontal axis bisecting this line at its midpoint) in each of 6 consecutive semicoronal slices and adds points for depth and intensity [26].

**Table 2:** MRI scoring methods

Disease activity assessed	Methodology
***Spondyloarthritis Research Consortium of Canada (SPARCC)***	
Inflammation in SI joints [26]	Each SI joint divided into 4 quadrants and scored for bone marrow edema in each of 6 consecutive semicoronal slices, with additional points for signal intensity and depth
Inflammation in spine [28]	Each discovertebral unit divided into 4 quadrants and scored in each of 3 slices for presence of bone marrow edema in each quadrant, with additional points for signal intensity and depth
	Total score: 0–414
***Canada-Denmark (CAN-DEN)***	
Inflammation and damage in spine [29]	The system is a detailed anatomy-based evaluation of inflammatory and structural lesions in vertebral bodies and posterior elements of the spine. Features assessed: inflammation (0–614), fat (0–510), bone erosion (0–208), new bone formation (0–460)
***Outcome Measures in Rheumatology (OMERACT) Psoriatic Arthritis MRI Scoring System (PsAMRIS)***^***a***^	
Inflammation and destructive changes in peripheral joints [30]	Features assessed (score range): • Synovitis (hands: 0–42; feet: 0–18) • Flexor tenosynovitis (hands: 0–42; feet: 0–18) • Periarticular inflammation (hands: 0–28; feet: 0–12) • Bone marrow edema (hands: 0–84; feet: 0–36) • Bone erosion (hands: 0–280; feet: 0–120) • Bone proliferation (hands: 0–14; feet: 0–6)

*SI* sacroiliac

^a^Exploratory analysis in a subset of patients (*n* = 50 in each group)

As exploratory assessments, MRIs will also be evaluated using the Canada-Denmark (CAN-DEN) score for spine MRI and the Outcome Measures in Rheumatology (OMERACT) Psoriatic Arthritis MRI Scoring System (PsAMRIS) score for MRI of the hands [30] and feet [31]. The CAN-DEN MRI spine scoring system evaluates inflammation, fat, bone erosion, and new bone formation of the spine in patients with spondyloarthritis. This system permits a detailed description of the involvement of different spinal structures, various topographic parts of the vertebral bodies, the facet joints, the spinous processes, the transverse processes, and the ribs and soft tissue. The system is designed for assessment of the individual types of MRI lesions and for acquiring total scores for the different types of lesions (inflammation, fat, erosion, and new bone formation). The OMERACT PsAMRIS score assesses MRI features (synovitis, tenosynovitis, periarticular inflammation, bone edema, bone erosion, and bone proliferation) in the hands and feet of PsA patients [29–31]. For this study, the investigator will select the most inflamed hand and the most inflamed foot; the selected hand and foot will be assessed by MRI at baseline, week 24, and week 52. MRI review for CAN-DEN and PsAMRIS will be undertaken by trained readers who have undergone prior calibration and will be blinded to the clinical data. Two readers are planned, with the utilization of a blinded adjudication reader for results that show a discrepancy between the two independent central readers during the screening and treatment phases.

Blood samples for genetic testing will be obtained from patients who provide additional consent. Samples will be collected before study intervention administration at visits when a study intervention administration is scheduled and will be used for pharmacogenomic analysis.

Guselkumab safety will be assessed through the frequency and type of AEs, serious adverse events (SAEs), AEs leading to discontinuation of study intervention, infections, and injection-site reactions. Malignancies and major adverse cardiovascular events will also be summarized. Adverse events will be reported by the patient (or, when appropriate, by a caregiver, surrogate, or the patient's legally acceptable representative) for the duration of the study.

### Study population

The target study population is patients with active, MRI-confirmed axPsA of the spine and/or SI joints. The planned enrolment is 135 patients per intervention group, for a total of 405 patients. To detect differences between each guselkumab group and placebo for the primary endpoint of change from baseline in BASDAI score at week 24, assuming a 2-sided alpha level of 0.05 and a power of > 99%, a sample size of 135 patients per treatment group was determined. Power calculations were performed utilizing a 2-sample *T*-test assuming equal variance for BASDAI change with mean (standard deviation [SD]) of − 1.28 (2.24), − 2.61 (2.47), and − 2.51 (2.00) for placebo, guselkumab 100 mg Q8W, and guselkumab 100 Q4W, respectively, based on observed mean changes from baseline in the DISCOVER 1 and 2 studies (data on file). The larger of the standard deviations between the guselkumab 100mg Q8W/Q4W and placebo groups was used, and the estimated effect sizes are 1.23 and 1.33 for guselkumab 100 mg Q4W and Q8W, respectively. No adjustments were made for multiplicity in the sample size calculations. Methods of patient recruitment will include referral networks, site patient databases, posters in hospitals and waiting rooms, and advertising.

Patient screening will be performed by the study investigator. Patients will be eligible for STAR if they have a diagnosis of PsA for ≥ 6 months, meet ClASsification criteria for Psoriatic ARthritis (CASPAR), and have active disease (≥ 3 swollen joints, ≥ 3 tender joints, and CRP ≥ 0.3 mg/dL), despite standard therapies (i.e., conventional synthetic disease-modifying anti-rheumatic drugs [csDMARDs], non-steroidal anti-inflammatory drugs [NSAIDs], or apremilast). A BASDAI score of ≥ 4, spinal pain component score ≥ 4, and MRI-confirmed axPsA (positive MRI spine and/or SI joints, defined as a SPARCC score ≥ 3 in either the spine and/or SI joints) are also required for study entry. Because there is currently no consensus defining MRI-confirmed axPsA, a SPARCC cutoff of ≥ 3 was selected by Steering Committee consensus. This was based on the established use of positive MRI to confirm axSpA [32], where the standard SPARCC cutoff is between ≥ 2 and ≥ 5 for the SI joints and ≥ 4 for the spine [33, 34]. A cutoff of ≥ 3 in either the spine and/or SI joints was judged to be adequately sensitive to the early signs of inflammation that distinguish axPsA.

Patients must also have current (plaque ≥ 2 cm) or a documented history of psoriasis. Patients with other inflammatory diseases and patients with any prior biologic DMARD or Janus kinase (JAK) inhibitor therapy,  as well as patients who have received apremilast within 4 weeks of study intervention, are not eligible. Concomitant use of stable doses of NSAIDs (≥ 2 weeks prior to first study agent administration); oral corticosteroids (equivalent to ≤ 10mg of prednisone/day for ≥ 2 weeks prior to first study agent administration); and one csDMARD, limited to methotrexate (MTX) (≤ 25 mg/week), sulfasalazine (≤ 3g/day), hydroxychloroquine (≤ 400 mg/day), and leflunomide (≤ 20 mg/day), will be permitted. Other key inclusion and exclusion criteria are provided in Table 3.

**Table 3:** Key inclusion and exclusion criteria

Inclusion criteria	Exclusion criteria
Age ≥ 18 years	Other inflammatory diseases (e.g., RA, AS, lupus)
Diagnosis of PsA for ≥ 6 months prior to enrolment and meet CASPAR criteria at screening	Previous biologic therapy
Active PsA: ≥ 3 swollen joints and ≥ 3 tender joints and CRP ≥ 0.3 mg/dL	Previous JAK inhibitor therapy
BASDAI ≥ 4	Prior therapy with systemic immunosuppressants; epidural, intra-articular, intramuscular, or intravenous (IV) corticosteroids, including adrenocorticotropic hormone; or apremilast within 4 weeks of study agent administration
Spinal pain VAS ≥ 4	Receiving ≥ 2 csDMARDs at baseline
Active plaque psoriasis (≥ 1 plaque of ≥ 2 cm and/or psoriatic nail changes) or documented history of psoriasis	
Imaging-confirmed (centrally read) PsA axial disease (positive MRI of spine and/or SI joints, defined as a SPARCC score of ≥ 3 in either the spine or the SI joints)	
Concomitant use of oral corticosteroids, NSAIDs, and/or one csDMARD was permitted at stables doses	
No history of latent or active tuberculosis	

*AS* ankylosing spondylitis, *BASDAI* Bath Ankylosing Spondylitis Disease Activity Index, *CASPAR* Classification criteria for Psoriatic Arthritis, *CRP* C-reactive protein, *csDMARD* conventional synthetic disease-modifying anti-rheumatic drug, *JAK* Janus Kinase, *MRI* magnetic resonance imaging, *NSAID* nonsteroidal anti-inflammatory drug, *PsA* psoriatic arthritis, *RA* rheumatoid arthritis, *SI* sacroiliac, *SPARCC* Spondyloarthritis Research Consortium of Canada, *VAS* visual analog scale

This study will be conducted in accordance with principles that originated in the Declaration of Helsinki, current International Conference on Harmonisation and Good Clinical Practice (GCP) guidelines, applicable regulatory requirements, and sponsor policy. The protocol and any modifications will be approved by the Institutional Review Board or Ethics Committee at each site and by local Health Authorities for each participating country. Investigators at each study site will collect written informed consent from all patients, with additional consent provided for voluntary genetic testing, prior to the conduct of any study-related procedures.

### Randomization and blinding

Central randomization will be implemented in this study to minimize bias in the assignment of patients to intervention groups, to increase the likelihood that known and unknown patient attributes (e.g., demographic and baseline characteristics) are evenly balanced across intervention groups, and to enhance the validity of statistical comparisons across intervention groups. Patients will be randomly assigned 1:1:1 to 1 of 3 intervention groups (guselkumab Q4W, guselkumab Q8W, or placebo with crossover to guselkumab Q8W at week 24) utilizing a computer-generated randomization schedule prepared before the study by or under the supervision of the sponsor. The interactive web response system will assign a unique intervention code, which will dictate the intervention assignment and matching intervention kit for the patient, who will be enrolled by the investigator at each study site. The randomization will be balanced by using randomly permuted blocks and will be stratified by csDMARD use (yes/no) and MRI peripheral (hands/feet) sub-study consent (yes/no). Of note, the MRI peripheral (hands/feet) sub-study will not be used in statistical analyses as a stratification factor; its sole purpose will be to balance the number of patients being assessed for MRI peripheral (hands/feet) among the 3 treatment groups.

Patients will receive a subcutaneous (SC) injection of guselkumab or placebo from the investigator at the study site at weeks 0 and 4. Beginning at week 8, the patient may administer study intervention at the study site under supervision. At week 32 and thereafter, patients may administer study intervention at home and on-site. The investigator will maintain a record of all study intervention dispensed to and returned by patients for home administration. Blinded intervention will be used to reduce potential bias during data collection and evaluation of clinical endpoints. To maintain the study blind, study intervention containers will be labeled only with the study name, intervention number, reference number, and storage instructions, and will not identify the study intervention itself. Data that may potentially unblind the intervention assignment (i.e., study intervention serum concentrations, anti-guselkumab antibody levels) will be handled with special care to ensure that the integrity of the blind is maintained and the potential for bias is minimized. The investigator will not be provided with patient randomization codes.

An independent joint assessor (IJA) will be designated at each study site to perform joint assessments (swollen and tender joint counts), as well as evaluations of enthesitis and dactylitis, and will be blinded to patient data. The IJA will have no other contact (other than joint assessments) with the patient once the patient is randomized, will not be the treating physician, will not discuss the patient’s clinical status with the patient or other site personnel during the joint assessment, and will not be permitted to review the patients’ medical records or the electronic case report form (eCRF) or any of the previous joint assessments.

At the week 24 database lock, the data will be unblinded to a limited number of sponsor personnel for analysis of the primary and major secondary endpoints (see Table 1) while patients are still participating in the study. Identification of sponsor personnel who will have access to the unblinded patient-level data will be documented prior to unblinding. Steering committee members, including Janssen employees, will not be unblinded prior to final database lock. No interim analyses are planned. Investigative study sites and patients will remain blinded to initial treatment assignment until after the final database is locked. Under normal circumstances, the blind should not be broken until all patients have completed the study and the database is finalized. The investigator, may in a medical emergency, determine the identity of the intervention by contacting the interactive web response system.

### Study design

**Fig. 2:**
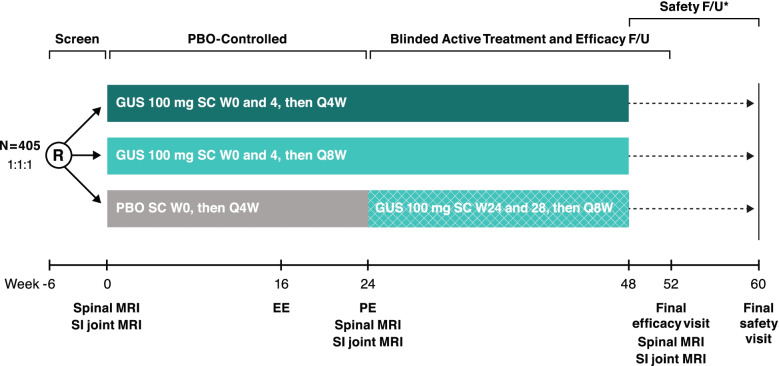
STAR study schema. Refer to Fig. 1 for study agent administration and dosing details. The asterisk (*) symbol indicates the following: 12-week safety follow-up (F/U) period begins at W48 after final study drug administration. EE, early escape; F/U, follow-up; GUS, guselkumab; MRI, magnetic resonance imaging; PBO, placebo; PE, primary endpoint; Q4W, every 4 weeks; Q8W, every 8 weeks; R, randomization; SC, subcutaneous; SI, sacroiliac; W, week

A target of 405 patients will be randomly assigned (1:1:1) in this study, with 135 patients planned per intervention group: SC guselkumab 100 mg Q4W, SC guselkumab 100 mg at week 0. week 4 and Q8W, or SC placebo with crossover at week 24 to SC guselkumab 100 mg Q8W (Fig. 2). Patients who meet early escape criteria (< 10% improvement from baseline in total back pain, for the purpose of this study assessed using BASDAI Question #2 and in morning stiffness measures as assessed by BASDAI Questions #5 and 6) at both week 12 and week 16 will be allowed to initiate or increase the dose of one permitted concomitant medication, up to the maximum allowed dose, at the investigator’s discretion.

The study comprises a screening phase of up to 6 weeks, a treatment phase of approximately 1 year that will include a placebo-controlled period from week 0 to week 24 and an active-controlled treatment period from week 24 to week 52 (last administration at week 48), and safety follow-up at week 60 (approximately 12 weeks after the last intended dose at week 48 per protocol).

### Intervention

Overall, the two guselkumab dose regimens demonstrated clinically meaningful efficacy and were well-tolerated with an acceptable safety profile in patients with active PsA in DISCOVER-1 and DISCOVER-2. This study is expected to provide additional clinical safety and efficacy data in patients with axPsA. Inclusion of both the 100 mg Q4W and Q8W dosing intervals will allow a relative benefit-risk assessment of both dose regimens.

The placebo control will be used to establish the frequency and magnitude of changes in clinical and imaging endpoints that may occur in the absence of active intervention. While guselkumab has been approved for patients with active PsA in several countries, the use of a placebo control is still necessary in the context of this study because the primary objective is to establish the efficacy of guselkumab for the treatment of axPsA for which there are limited data.

Guselkumab 100 mg and matching liquid placebo for guselkumab will be provided in single-use prefilled syringes assembled with the Ultrasafe PLUS^TM^ Passive Needle Guard. Study intervention should be administered under the supervision of the investigator or a qualified study site personnel. Patients will have the option to self-administer the last 3 doses of the study medication at home. Therefore, drug accountability will be important for adherence.

Patients who discontinue study intervention for any reason will be encouraged to continue in the study by returning for all remaining study visits. For patients who discontinue study intervention prior to week 24, MRIs of the SI joint, spine, and hands and feet (if consented separately) should be performed at the time of study intervention discontinuation. In addition, MRIs should be repeated at week 24 if discontinuation occurs prior to or at week 16. If a patient discontinues study intervention at or after week 24, but prior to the week 52 visit, the final efficacy visit should occur at the time of discontinuation or as soon as possible thereafter and all assessments under the week 52/final efficacy visit should be performed with the exception of study intervention administration. In either scenario, the patients will be instructed to return for a final safety visit to perform assessments under the week 60/final safety visit approximately 12 weeks after the last study intervention administration.

### Statistical methods

Descriptive statistics, such as mean, SD, median, interquartile range, minimum, and maximum for continuous variables and counts and percentages for discrete variables, will be used to summarize most efficacy data. For binary response endpoints, treatment comparisons (difference versus placebo with 95% CI) will generally be performed using a chi-square test or a Cochran-Mantel-Haenszel test. For continuous endpoints, treatment comparisons will be performed using an analysis of covariance, a mixed model for repeated measures (MMRM) or a constrained longitudinal data analysis model. In general, statistical testing will be performed using 2-sided tests. The overall type I error of the treatment comparisons of both dose regimens versus placebo for the primary and the 5 selected major secondary endpoints (Table 1) will be controlled at a significance level of ≤ 0.05. The power calculations for the key study endpoints with *N* = 135 per treatment group and a 1:1:1 randomization ratio are based on a 2-sided significance level of 0.05 using a 2-sample *T*-test, assuming equal variances for continuous variables and testing 2 proportions using the *Z*-test with pooled variance for binary variables. Sensitivity analyses will be performed for some endpoints. Treatment group comparisons will not be performed after week 24 when patients in the placebo group will cross over to guselkumab.

The primary endpoint, change from baseline in BASDAI score at week 24, will be analyzed based on the adjusted composite estimand defined by 5 components: population, treatment, variable (endpoints), intercurrent events, and population level summary. In addition to the total BASDAI composite score, change from baseline in spinal pain (Question #2) by visit over time through week 52 will be evaluated independently. The change from baseline in the BASDAI score will be compared between each guselkumab group and the placebo group for all patients and will be carried out on the full analysis set defined by all randomized patients who received at least one partial or full administration of study intervention. The MMRM, which relies on the missing at random (MAR) assumption for the missing data, will be used to test the difference between each guselkumab group and the placebo group. Under the assumption of MAR, missing data will be accounted for through correlation of repeated measures in the model. Explanatory variables of the MMRM model will include treatment group, visit, baseline BASDAI score, csDMARD use (Yes/No), baseline HLA-B27 status (positive/negative), an interaction term of visit with treatment group, and an interaction term of visit with baseline BASDAI score. Treatment difference of change from baseline in BASDAI score at week 24 between each guselkumab group and the placebo group will be provided by the difference in the least squares means (LSmeans). The 95% CI for the differences in LSmeans and *p*-values will be calculated based on the MMRM. An unstructured covariance matrix for repeated measures within a patient will be used. The *F*-test will use Kenward-Roger’s approximation for degrees of freedom.

Patients meeting treatment failure criteria, i.e., patients who discontinue study intervention due to any reason except due to study conduct affected by COVID-19, who initiated or increased the dose of csDMARDs or oral corticosteroids from baseline for treatment of PsA, or who initiated protocol prohibited medications/therapies for PsA prior to week 24, will be considered nonresponders for binary endpoints or will be assumed to be MAR for continuous endpoints in the MMRM (except for the MRI endpoint, which will utilize multiple imputation). Through week 24, observed data from patients who discontinue study intervention due to study conduct affected by COVID-19, or who exhibit substantial treatment non-compliance due to study conduct affected by COVID-19, will be assumed to be MAR.

In addition to the primary endpoint, five major secondary endpoints have been identified as important to assess different attributes of the disease, and only these variables will be multiplicity controlled (Table 1). The overall type I error of the treatment comparisons of both guselkumab dosing regimens versus placebo for the primary and the 5 selected major secondary endpoints will be controlled at a significance level of ≤ 0.05. For these pre-specified primary and secondary endpoints, both adjusted and nominal (unadjusted) *p*-values will be provided. In the instance that an adjusted *p*-value is not significant, the nominal (unadjusted) *p*-value must only be interpreted as supportive. All other secondary endpoints will be summarized over time by treatment groups, with treatment comparisons performed by visit through week 24 as detailed in the statistical analysis plan.

Subgroup analyses will be performed to evaluate consistency in the primary efficacy endpoint by demographic characteristics, baseline disease characteristics, and baseline medications. Interaction testing between the subgroups and treatment group will also be provided if appropriate.

For the major secondary endpoints of change from baseline at week 24 in SPARCC score for MRI SI joints and for MRI spine, change from baseline for the outcomes assessed will be calculated among patients with a positive MRI of SI joints and spine, respectively, at baseline. Analyses of other major secondary endpoints will include calculating the proportion of patients achieving a BASDAI50 response, as well as the proportion of patients with IGA 0/1 response at week 24 among the patients with ≥ 3% body surface area psoriatic involvement and an IGA score of ≥ 2 (at least mild) at baseline. The proportions of patients achieving clinically important improvement in ASDAS (change of ≥ 1.1), major improvement in ASDAS (change of ≥ 2.0), ASDAS inactive disease (score < 1.3) [35], and ASDAS low disease activity (score < 2.1) will be calculated [36]. Change from baseline in CAN-DEN score for MRI spine will be assessed among patients with baseline CAN-DEN score ≥ 3.

All safety analyses will be performed using the Safety Population, i.e., all patients who receive ≥ 1 study agent administration. Analyses of AEs used to assess the safety of guselkumab will include the incidence and type of AEs, SAEs, infections, and injection site reactions. Laboratory data will be summarized by type of laboratory test; descriptive statistics will be calculated for selected laboratory analytes at baseline and for observed values and changes from baseline at each scheduled timepoint. Vital signs including pulse/heart rate and blood pressure (systolic and diastolic) will be summarized over time, using descriptive statistics and/or graphically. The proportion of patients with values beyond clinically important limits will be summarized.

### Oversight and monitoring

A Trial Steering Committee of independent members has been created for study consultation purposes. Steering committee objectives are to (1) provide practical advice on strategy and direction of the trial; (2) provide clinical expertise and advice on best clinical study parameters (program design, population, endpoints, etc.); (3) participate in data review, analysis, and interpretation of the result from the trial; and (4) guide/suggest important analyses to inform clinical practice. As part of study oversight, sponsor personnel will monitor study site conduct to ensure the protocol and GCP are followed.

### Frequency and procedures for auditing trial conduct

To ensure accuracy and reliability of data, qualified investigators and appropriate study sites have been selected for this study. Protocol procedures and eCRF guidelines have been reviewed with investigators and study site personnel, and clinical laboratory data will be transmitted directly to the sponsor’s database and verified for accuracy and consistency. Representatives of the sponsor’s clinical quality assurance department may visit the study site at any time during or after completion of the study to conduct an audit of the study in compliance with regulatory guidelines and company policy to review study records. The sponsor will also review the eCRF for accuracy and completeness, and discrepancies will be resolved with the appropriate investigator.

Patient privacy guidelines and applicable laws will be adhered to. Similar auditing procedures may also be conducted by agents of any regulatory body, either as part of a national GCP compliance program or to review the results of this study in support of a regulatory submission.

## Discussion

A post hoc analysis of pooled data from the Phase 3 DISCOVER-1 and DISCOVER-2 studies indicated that treatment with guselkumab improved axial symptoms in patients with PsA who had investigator and imaging-confirmed sacroiliitis. STAR, a phase 4, prospective, multicenter, randomized clinical trial, will now allow for an in-depth evaluation of the efficacy and safety of selectively inhibiting the IL-23p19 subunit with guselkumab in patients with MRI-confirmed axPsA. MRIs of the SI joints and spine in STAR will be centrally read, with readers blinded to treatment group and timepoint, using methods specifically designed to assess axial inflammation.

AxPsA as a unique presentation is supported by differing responses among patients with axSpA to biological agents that target the IL-23/IL-17 axis. Despite the efficacy of IL-12/23 and IL-23 inhibitors in PsA [9, 16–18] and the efficacy of IL-17 inhibitors in PsA and axSpA [37, 38], a trial of an IL-23 inhibitor, risankizumab, in AS demonstrated negative results [39]. Additionally, cumulative evidence from three phase 3 placebo-controlled trials of patients with axSpA showed that patients treated with the anti-IL-12/23p40 monoclonal antibody ustekinumab did not achieve clinically meaningful improvement across key efficacy endpoints when compared with placebo [15]. In contrast, significant improvements in axial signs and symptoms of PsA were demonstrated in two phase 3, multicenter, double-blind, placebo-controlled studies that evaluated the efficacy and safety of ustekinumab [9]. A subsequent analysis from these same PsA studies, which focused on spondylitis-related endpoints in tumor necrosis factor inhibitor (TNFi)-naïve patients with peripheral arthritis and physician reported-spondylitis, found that ustekinumab demonstrated clinically meaningful changes across BASDAI measures of neck/back/hip pain, as well as the modified BASDAI (omission of Question #3, peripheral joint pain or swelling), when compared with placebo. From this, it was concluded that ustekinumab has the potential to improve disease activity in TNFi-naïve PsA patients with axial involvement [40]. The pathophysiology of AS and axPsA might indicate two different diseases. There has, for example, been discussion of biologic mechanisms in the spine that differ from peripheral joints and entheses, such as IL-23-independent production of IL-17 [41, 42]. Additionally, a recent analysis of AS patients receiving ustekinumab and axPsA patients receiving guselkumab showed that AS and axPsA patients have different genetic risk factors and serum IL-17 levels [43]; differential expression of bone biomarkers between patients with AS and those with axPsA has also been reported in a cohort of cases of PsA without axial arthritis, psoriatic spondyloarthritis, and AS [44].

The lack of a consensus definition for classifying axPsA, as well as validated instruments for assessing response to treatment, represent a significant unmet need. Ongoing initiatives with Assessment of SpondyloArthritis international Society (ASAS) and the Group for Research and Assessment of Psoriasis and Psoriatic Arthritis (GRAPPA), including a study of patients with PsA that focuses on inflammatory changes in the axial skeleton as assessed by imaging (radiograph and MRI), may inform efforts to prospectively develop such criteria [45, 46]. Currently, assessments of axial symptoms in patients with PsA rely primarily upon instruments designed originally for patients with AS. For example, in the only existing dedicated prospective randomized clinical trial that has evaluated axPsA using imaging assessments, biologic-naïve adults with PsA were eligible if they showed symptoms of active spinal disease, which were defined as a BASDAI score ≥ 4 and spinal pain score ≥ 40 (0–100 mm VAS) despite NSAID therapy. In part, efficacy was assessed by MRI of the spine and SI joint using Berlin scoring methodology. However, imaging-confirmed axPsA, whether by radiograph or MRI, was not among the study inclusion criteria. While results indicated significant improvement across imaging endpoints, lack of agreement surrounding the imaging criteria used to define axPsA, as well as the limited translatability of criteria used for AS in assessing axPsA, were noted as study limitations. The absence of a definition for axPsA, including imaging criteria, was also noted as a limitation in another study that evaluated the efficacy of secukinumab, a monoclonal antibody that directly inhibits IL-17A, in patients with PsA, where exploratory analyses of ASAS and BASDAI responses were not significantly influenced by MRI status at baseline [14]. Thus, a better understanding of axPsA is needed, as are effective therapies to improve axial symptoms and reduce axial inflammation as objectively assessed by imaging.

Notably, the present study introduces new methods of MRI assessment that can advance the understanding of how imaging is used to evaluate axPsA. The imaging needs of the present study required using estimations based on prior axSpA studies [32–34] to develop a SPARCC scoring method for MRI that would best capture the inflammation levels specific to axial involvement. Additionally, this study will be the first time that CAN-DEN, traditionally used for patients with spondyloarthritis, will be used to assess axPsA. With the unique application of these scoring methods, the results of the present study will offer valuable information about the classification and outcome measures of axPsA.

Efforts to define disease and outcomes in axPsA, and to advance treatment recommendations, are ongoing [8, 10, 40, 46–48]. Differences in the etiology of axial inflammation between axSpA and PsA might require a different treatment approach; thus, an increased understanding of axPsA has the potential to improve the treatment options available for patients. Research supports the necessity of SI and spinal imaging in detecting axPsA; however, data surrounding differences in axial involvement using MRI are limited [10]. The STAR study will provide an opportunity to demonstrate that the selective IL-23 inhibitor guselkumab not only significantly relieves the symptoms of axPsA but also decreases axial inflammation as shown by MRI.

## Trial status

The first patient was screened on 30 August 2021, and the last patient out is expected on 10 February 2024. Protocol version 1.0, 14 April 2021.

## Supplementary Information

**Additional file 1.** STAR Trial Registration Data.

**Additional file 2. **STAR Trial SPIRIT Checklist.

## Data Availability

Data will be available according to the data sharing policy of Janssen Pharmaceutical Companies of Johnson & Johnson (https://www.janssen.com/clinical-trials/transparency). Trial results will be posted online as required by ClinicalTrials.gov, and results will be shared with patients as required by local regulations.

## Abbreviations

AE: Adverse event
AS: Ankylosing spondylitis
ASAS: Assessment of SpondyloArthritis international Society
ASAS40: ≥40% improvement in Assessment of SpondyloArthritis international Society response criteria
ASDAS: AS Disease Activity Score utilizing C-reactive protein
axPsA: Axial psoriatic arthritis
axSpA: Axial spondyloarthritides
BASDAI: Bath Ankylosing Spondylitis Disease Activity Index
BASDAI50: ≥50% improvement in Bath Ankylosing Spondylitis Disease Activity Index score
CAN-DEN: Canada-Denmark
CASPAR: Classification criteria for Psoriatic ARthritis
CRP: C-reactive protein
csDMARD: Conventional synthetic disease-modifying anti-rheumatic drug
CTCAE 5.0: Common Terminology Criteria for Adverse Events
DAPSA: Disease Activity Index for Psoriatic Arthritis
eCRF: Electronic case report form
EE: Early escape
F/U: Follow-up
GCP: Good Clinical Practice
GRAPPA: Group for Research and Assessment of Psoriasis and Psoriatic Arthritis
HAQ-DI: Health Assessment Questionnaire – Disability Index
HLA: Human leukocyte antigen
ICH: International Conference on Harmonisation
IGA: Investigator’s global assessment
IJA: Independent joint assessor
IL: Interleukin
IV: Intravenous
JAK: Janus kinase
LS: Least squares
MAR: Missing at random
MDA: Minimal disease activity
MMRM: Mixed model for repeated measures
MRI: Magnetic resonance imaging
NSAID: Nonsteroidal anti-inflammatory drug
OMERACT: Outcome Measures in Rheumatology
PK: Pharmacokinetics
PsA: Psoriatic arthritis
PsAMRIS: Psoriatic Arthritis MRI Scoring System
Q4W: Every 4 weeks
Q8W: Every 8 weeks
R: Randomization
RA: Rheumatoid arthritis
SAE: Serious adverse event
SC: Subcutaneous
SD: Standard deviation
SI: Sacroiliac
SPARCC: Spondyloarthritis Research Consortium of Canada
TNFi: Tumor necrosis factor inhibitor
VAS: Visual analog scale
