# Supplementary material for: Efficacy and safety of guselkumab in biologic-naïve patients with active axial psoriatic arthritis: study protocol for STAR, a phase 4, randomized, double-blinded, placebo-controlled trial
Source: Trials. 2022 Sep 5;23:743. doi: 10.1186/s13063-022-06589-y (PMC9444112; doi:10.1186/s13063-022-06589-y)
Supplement: Supplementary file 1 — Additional file 1. STAR Trial Registration Data. [file 13063_2022_6589_MOESM1_ESM.docx]

| **Additional File 1. STAR Trial Registration Data** | |
| --- | --- |
| Data category | Information |
| Primary registry and trial identifying number | ClinicalTrials.gov  NCT04929210 |
| Date of registration in primary registry | 18, June 2021 |
| Secondary identifying numbers | CR109043, 2021-000465-32,  CNTO1959PSA4002 |
| Source of monetary or material support | Janssen Scientific Affairs, LLC |
| Contact for public queries | Phone: 844-434-4210  Email: [Participate-In-This-Study@its.jnj.com](mailto:Participate-In-This-Study@its.jnj.com) |
| Contact for scientific queries | Phone: 844-434-4210  Email: [Participate-In-This-Study@its.jnj.com](mailto:Participate-In-This-Study@its.jnj.com) |
| Public title | A Study of Guselkumab Administered Subcutaneously in Bio-naive Participants With Active Psoriatic Arthritis Axial Disease (STAR) |
| Scientific title | A Phase 4, Multicenter, Randomized, Double-blind, Placebo-controlled Study Evaluating the Efficacy and Safety of Guselkumab Administered Subcutaneously in Bio-naive Participants With Active Psoriatic Arthritis Axial Disease |
| Countries of recruitment | United States of America, Argentina, Australia, Bulgaria, Canada, the Czech Republic, Germany, Hong Kong, Hungary, Israel, Italy, Malaysia, Poland, Portugal, Russian Federation, Spain, Taiwan, Turkey, Ukraine, United Kingdom |
| Health condition(s) or problem(s) studied | Psoriatic arthritis |
| Intervention(s) | Active comparator: 100 mg subcutaneous guselkumab Q4W or Q8W  Placebo comparator: matching liquid placebo for guselkumab is subcutaneous saline Q4W through W20 |
| Key inclusion and exclusion criteria | Ages eligible for study: 18 years and older  Sexes eligible for study: All  Accepts healthy volunteers: No  Inclusion criteria: See Table 3  Exclusion criteria: See Table 3 |
| Study type | Interventional  Allocation: Randomized, parallel assignment intervention model, double-blind masking (participant, investigator)  Primary purpose: Treatment  Phase 4 |
| Date of first enrollment | 1, December 2021 |
| Target sample size | 405 participants |
| Recruitment status | Recruiting |
| Primary outcome(s) | See Table 1 |
| Key secondary outcomes | See Table 1 |
